# Assesment of Functional and Radiological Outcomes of Proximal Tibia Fractures Surgically Treated With Ilizarov Fixators

**DOI:** 10.7759/cureus.79464

**Published:** 2025-02-22

**Authors:** Gyandeep Sarangi, Spandan Mishra, Tapan K Das, Ramanuj Acharya, Abhinav Sharma

**Affiliations:** 1 Orthopaedics and Traumatology, Institute of Medical Sciences and SUM Hospital, Bhubaneswar, IND

**Keywords:** fracture union, ilizarov fixator, koos score, proximal tibia fracture, shatzker classicification

## Abstract

Introduction and objectives

Ilizarov ring fixators have long been used in the case of shaft fractures of long bones and infected non-union. The use of primary Ilizarov ring fixators in an intra-articular fracture has always been controversial and has its own set of requirements and challenges. We wanted to analyse the functional and radiological outcome of proximal tibia fractures and look for any complications that may develop during the postoperative follow-up period.

Materials and methods

This was a prospective observational study conducted on patients operated upon at a tertiary care centre in Odisha, India. This study included patients aged 16-70 years who had presented to the emergency or the outpatient department with displaced proximal tibia fractures of Shatzker type three to six. Exclusion criteria included patients with floating knee, pathological fractures of proximal tibia, undisplaced proximal tibia fractures, patients beyond the age limits, congenital deformities, unwilling patients, patients managed conservatively for other medical reasons, and patients with neurovascular deficits following the trauma. The functional scores used for the same were the Knee injury and Osteoarthritis Outcome Score (KOOS) and Association for the Study and Application of the Methods of Ilizarov (ASAMI) score and Rasmussen Radiological Score (RRS) was used for the radiological assessment of fracture site union. The participants were followed up at three weeks, six weeks, three months, six months, and 12 months.

Results

The patients had an average KOOS of 74.9 with the majority in the range of 75-100. The majority of the cases had an excellent ASAMI score. The RRS was found to be good in the majority of cases. All the patients were followed up for 12 months and an average of 7.4 months was observed for radiological union and Ilizarov frame removal.

Conclusion

Ilizarov ring fixator is an excellent device to aid in the union of the fractured fragments in case of proximal tibia fractures. This method aids in early weight bearing. It is a very versatile device and can also be used as a salvage procedure. However, the application of an Illizarov fixator has a slow learning curve. Excellent results can be achieved using an Ilizarov fixator by understanding its biomechanical properties and its principles.

## Introduction

Intra-articular proximal tibia fractures are common lower limb fractures due to their superficial position and a steep increase in high-velocity traumas, primarily due to road traffic accidents [1,2]. Metaphyseal fractures of the intra-articular kind in the case of the proximal tibia, primarily occur due to direct external force injury resulting in direct bending forces at the site of injury. Usually, a combination of valgus, varus and axial forces act on the proximal tibia. High-energy trauma to the proximal tibia is usually associated with soft tissue injuries, while low-energy trauma is usually associated with lateral tibia plateau fracture.

Although many methods of anatomical fracture site fixation are currently available that produce excellent results, each method has its own advantages and disadvantages, accompanied by a certain set of indications and contra-indications [3]. Fracture site fixation using Ilizarov external ring fixators has been used since the days of World War 2 for shaft fractures of long bones and infected non-unions [4]. The use of Ilizarov external ring fixators for anatomical reduction of the fracture site in intra-articular fractures has been quite controversial and does present with its own set of challenges. Although the risk of osteomyelitis and intraoperative loss of large amounts of blood is quite low with good soft tissue healing and early weight bearing, a perfect or near-perfect anatomical reduction at the fracture site is quite difficult to achieve, which has quite often been a deciding factor for the use of Ilizarov external ring fixators [5,6], apart from the factor of patient’s cosmesis. The problem of superficial pin tract infection, demanding regular daily or bi-daily attention also often limits the patient’s compliance.

Although a few studies have been done, there has not been much evidence to bring forth the efficiency of Ilizarov external ring fixators in the surgical management of intra-articular proximal tibia fractures. This study was undertaken to evaluate the functional and radiological scores in proximal tibia fractures of Shatzker type three to six in patients surgically treated with Ilizarov external ring fixators.

## Materials and methods

This was a prospective observational study conducted from 2022 to 2024 at the Department of Orthopaedics of the Institute of Medical Sciences (IMS) and SUM Hospital, a tertiary hospital in Bhubaneswar, Odisha, India. The study was approved by the Institutional Ethics Committee, IMS and SUM Hospital (approval number: IEC/IMS.SH/SOA/2024/682).

Inclusion and exclusion criteria

The inclusion criteria were patients between the ages of 18 and 70 years with a proximal tibia fracture of Shatzker type three to six undergoing surgical fixation with an Ilizarov ring fixator. Patients with a floating knee, pathological fracture of the proximal tibia, un-displaced proximal tibia fractures, congenital deformities of the proximal tibia, and distal neurovascular deficit and patients managed conservatively due to other medical conditions were excluded from the study, and the same was noted at this stage for prognostic and follow-up purposes.'

Patient selection

Thus, all the patients aged 16-70 years who had presented to the orthopaedic outpatient department and the emergency department with injury to the knee were advised to take a digital radiograph of the affected knee in anteroposterior and lateral views. Patients having a proximal tibia fracture of Shatzker type three to six were segregated. Among these, the patients who were preoperatively planned for surgical fixation with Ilizarov ring fixators were given a detailed explanation of the study. The patients who then volunteered themselves for the same were included in the study after taking their informed consent.

A total of 30 cases were included in the study as per the inclusion and exclusion criteria.

Procedure

The cross-sectional anatomy and hence the safe corridors of the leg were identified and marked to avoid damage to the neuro-vascular structures. Kirschner wire (K wire) of cross-sectional diameter of 1.8 mm with either a trochar tip or Bayonet tip for metaphyseal and diaphyseal areas of the long bone, respectively, was used. Prior to the placement of the K wires, the muscles and skin were maximally stretched at the adjacent joints. The K wires were inserted from a more vulnerable site to a relatively less vulnerable site and drilling was only done after reaching the bone surface. Periodic stopping of drilling inside the bone with the use of a saline-soaked gauze piece was done to prevent heating of the bone and hence pin loosening and infection due to heat necrosis. In case of undue tension at the K wire insertion site, the K wire was withdrawn to the level of the skin, re-adjusted, and then proceeded so that the skin was in a better position. In case of any vascular injury, drilling of the K wire was stopped and local pressure was applied using a gauze piece to stop the bleeding. A new entry was then done for K wire insertion. Distal pulses were always checked. For maximal stability of the construct, the K wires were placed perpendicular to each other and at least 0.5 mm apart. The K wire ends were then tensioned using a dynamometer tensioner in all the cases. Corticotomy was done in eight cases due to acute bone loss.

In the postoperative day one, the patients were allowed to perform static and dynamic quadriceps strengthening exercises. Active and passive knee range of motion (ROM) exercises were performed under the supervision of a physiotherapist. They were also made to bear weight on the affected limb using a universal walker. In the cases where a corticotomy was performed, a latent period of 10 days was taken on average and a distraction was then done at the rate of one mm per day in four divided increments. The initial distraction was done by the treating surgeon and demonstrated to the patient’s relatives. The further distractions were done under supervision by the patient’s relatives or the patient until discharge. The Ilizarov ring construct was kept clean by wiping it with hydrogen peroxide and betadine solution by the patient and the patient’s relatives.

The patients included in the study were followed up at three weeks, six weeks, three months, six months, and 12 months. Any problems or obstacles and issues that occurred during the treatment period were managed or treated as per the respective requirements.

The Ilizarov frame removal was decided as per the radiological union of the fracture site and regenerated new bone formation. The new bone formation was remodelled with the medullary canal and cortex of almost equal or similar diameter as that of the host bone corticotomy surfaces prior to the Ilizarov ring frame removal.

The ring frame removal was initiated by dynamization or loosening the nuts of the frame and allowing the patient to walk on full weight bearing with the attached frame. If the patient had no pain, then they were allowed to ambulate for 15 days with the loosened frame attached. The frame was then removed with a patellar tendon bearing cast, which was applied for one month.

Study tools

The Knee injury and Osteoarthritis Outcome Score (KOOS) [7] and the Association for the Study and Application of the Method of Ilizarov (ASAMI) score (see Appendices) were used for the functional evaluation, while the Rasmussen Radiological Score (RRS) [8] was used for radiological evaluation.

## Results

Most of the cases of proximal tibia fractures in the study were due to road traffic accidents (n=18; 60%), followed by accidental falls and other causes, both of which accounted for six cases each (20% each). Male participants (n=16; 53%) were higher in number as compared to female participants (n=14; 46%) with a combined mean age of 41.62 years, as shown in Table 1.

**Table 1:** Demographic variables of the study patients (N=30)

	Age (years)	Road traffic accidents	Accidental falls	Others
Male (n=16)	37.5	12 (40%)	2 (6%)	2 (6%)
Female (n=14)	45.75	6 (20%)	4 (13%)	4 (13%)

The cases taken up for the Ilizarov ring fixator were compound cases of Gustelo-Anderson type one, two, and three as shown in Table 2.

**Table 2:** Distribution of the cases as per the Gustelo-Anderson classification (N=30)

Type	Number of cases	Percentage
Grade I	6	20%
Grade II	8	26%
Grade III	16	53%

As far as the comorbid condition of the patients was concerned, six participants were known to have type 2 diabetes mellitus, eight had hypertension, and four had other co-morbidities.

On regular follow-ups of the patients, it was seen that fracture site union was achieved in 29 cases (96.6%), of which three (9.9%) cases had a mal-union and one (3.3%) case had an infected non-union which required further surgical intervention. Pin tract infection was observed in 10 (33.3%) cases and premature consolidation of the corticotomy site was observed in two (6.6%) cases. One case (3.3%) developed a persistent infection, which later caused an infected non-union at the fracture site. This has been shown in Table 3.

**Table 3:** Postoperative complications in the study participants (N=30)

Complications	Frequency (Percentage)
Premature consolidation of cortocotomy site	2 (6.66%)
Pin tract infection	10 (33.33%)
Infected non-union	1 (3.33%)
No complications	17 (56.66%)

The mean functional and radiological scores were calculated. The mean KOOS score was observed to be 78.64±6.66, and the mean ASAMI on average was found to be good. On radiological score calculation, the mean score was observed to be 14.33±2 at the end of the 12-month follow-up. The tabular evaluation is shown in Table 4.

**Table 4:** Comparision of scores KOOS: Knee injury and Osteoarthritis Outcome Score; ASAMI: Association for the Study and Application of the Method of Ilizarov; RRS: Rasmussen Radiological Score

Score	Value
KOOS	78.64±6.66
ASAMI	Good
RRS	14.33±2

A total of 24 (80%) patients had an excellent outcome, four (13.34%) had a good outcome, and two (6.66%) had an unfavourable or poor outcome, as shown in Table 5.

**Table 5:** Distribution of participants according to outcomes (N=30)

Outcome	Frequency (Percentage)
Excellent	24 (80%)
Good	4 (13.34%)
Poor	2 (6.66%)

## Discussion

Proximal tibia fractures present with a varied spectrum of soft tissue bony injuries which could lead to permanent disability if not treated properly. Although the causes of proximal tibia fractures are numerous, high-velocity traumas such as road traffic accidents top the list. The incidence and prevalence of proximal tibia fractures are on the rise due to an increase in road traffic accidents and, hence, it has become increasingly crucial to properly address a proximal tibia fracture to improve the quality of life and prevent morbidity. Displaced proximal tibia fractures produce an unsatisfactory result when treated conservatively because of major limitations of inadequate reduction and ineffective fracture site alignment [9-11].

Conventionally treated with open reduction and internal fixation and plating, an Ilizarov ring fixator is a very well acceptable alternative which, under fluoroscopy guidance, provides for good anatomical reduction and fixation with minimal soft tissue damage, hence leading to a good functional and radiological outcome [12-14]. Although an external ring fixator prevents further soft tissue damage, there are possible concerns of pin tract infection, malunion, and poor patient compliance due to cosmesis [15].

Our study mostly had young adults with a mean age of 41.62 years. Duwelius and Connolly [16] reported an average of 48 years and Porter [17] reported an average of 47 years as the average demography, which are in the similar average age observation of our study. There was a higher number of male patients, indicating a probable male preponderance of proximal tibia fractures. This is a finding similar to that reported by Albuquerque et al. [18], Manidakis et al. [19], and Mehin et al. [20].

In the current study, pin tract infection was observed in 33.33% of cases, which is more than that reported by Babis et al. (9%) [21]. The average active knee flexion is 120°, whereas, in the current study, the postoperative knee flexion was observed to be 110±20° at the six-month follow-up.

Procedure

**Figure 1:**
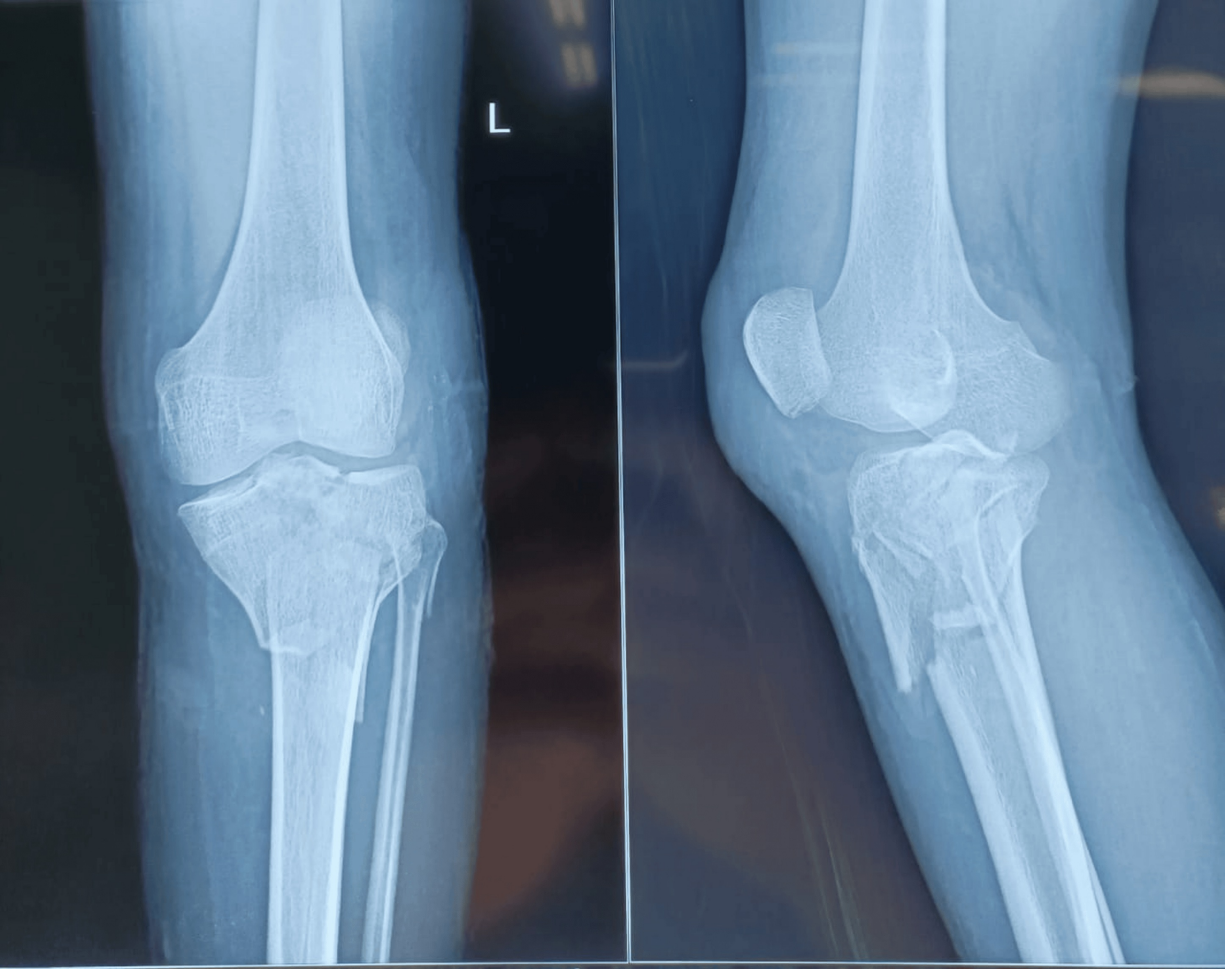
Preoperative X-ray picture showing Shatzker type 6 proximal tibia fracture

The preoperative evaluation was done as shown in Figure 1.

**Figure 2:**
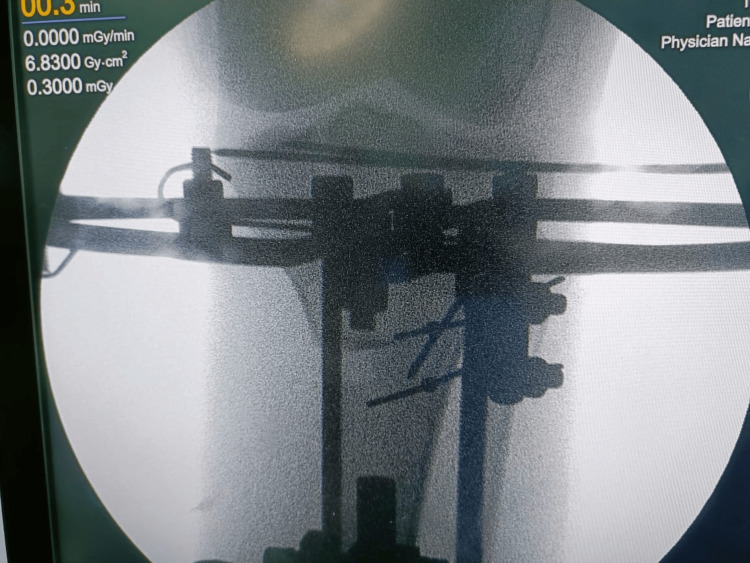
Intra-operative C-arm Image after an acceptable reduction and Ilizarov placement

Intraoperatively, using C-arm images, reduction was achieved and the Ilizarov frame was applied. The reduction was done as shown in Figure 2.

**Figure 3:**
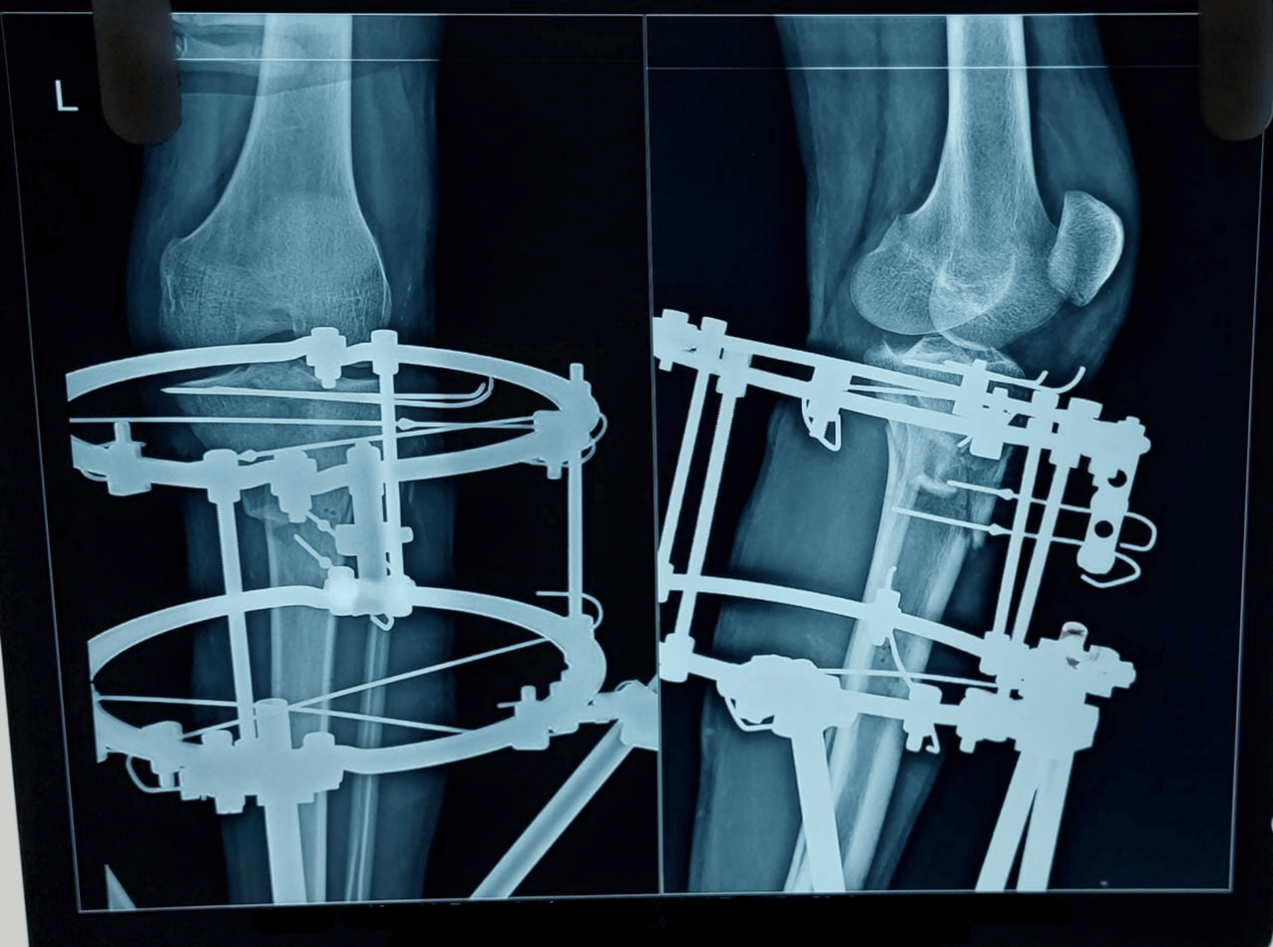
Postoperative X-ray after reduction and Ilizarov construct placement

The mean surgical time was observed to be 43.0±16 minutes, with a postoperative hospital stay of five days in patients without corticotomy and 15 days in case of patients with corticotomy where, after 10 days, they were made to distract the fracture site under supervision as shown in Figure 3.

**Figure 4:**
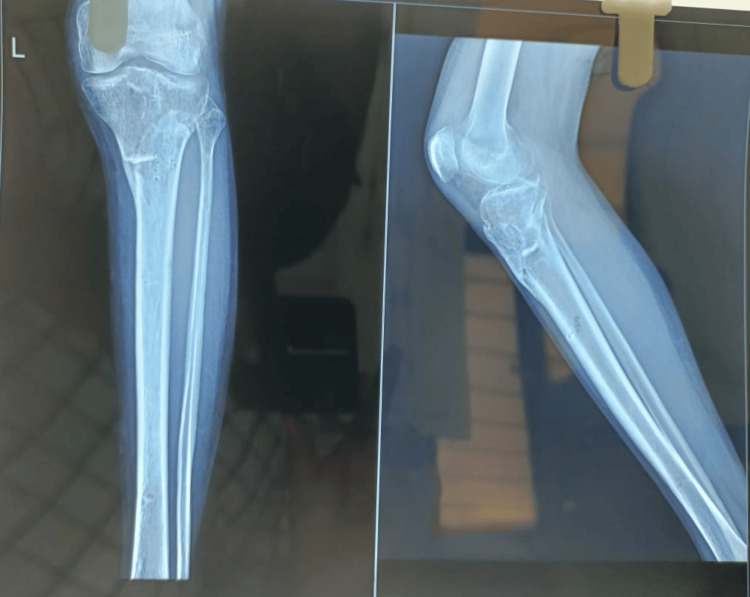
X-ray after removal of Ilizarov construct after achieving radiological union at the fracture site

An average of 7.4 months was observed for radiological fracture site union and Ilizarov ring external fixator removal, as shown in Figure 4.

**Figure 5:**
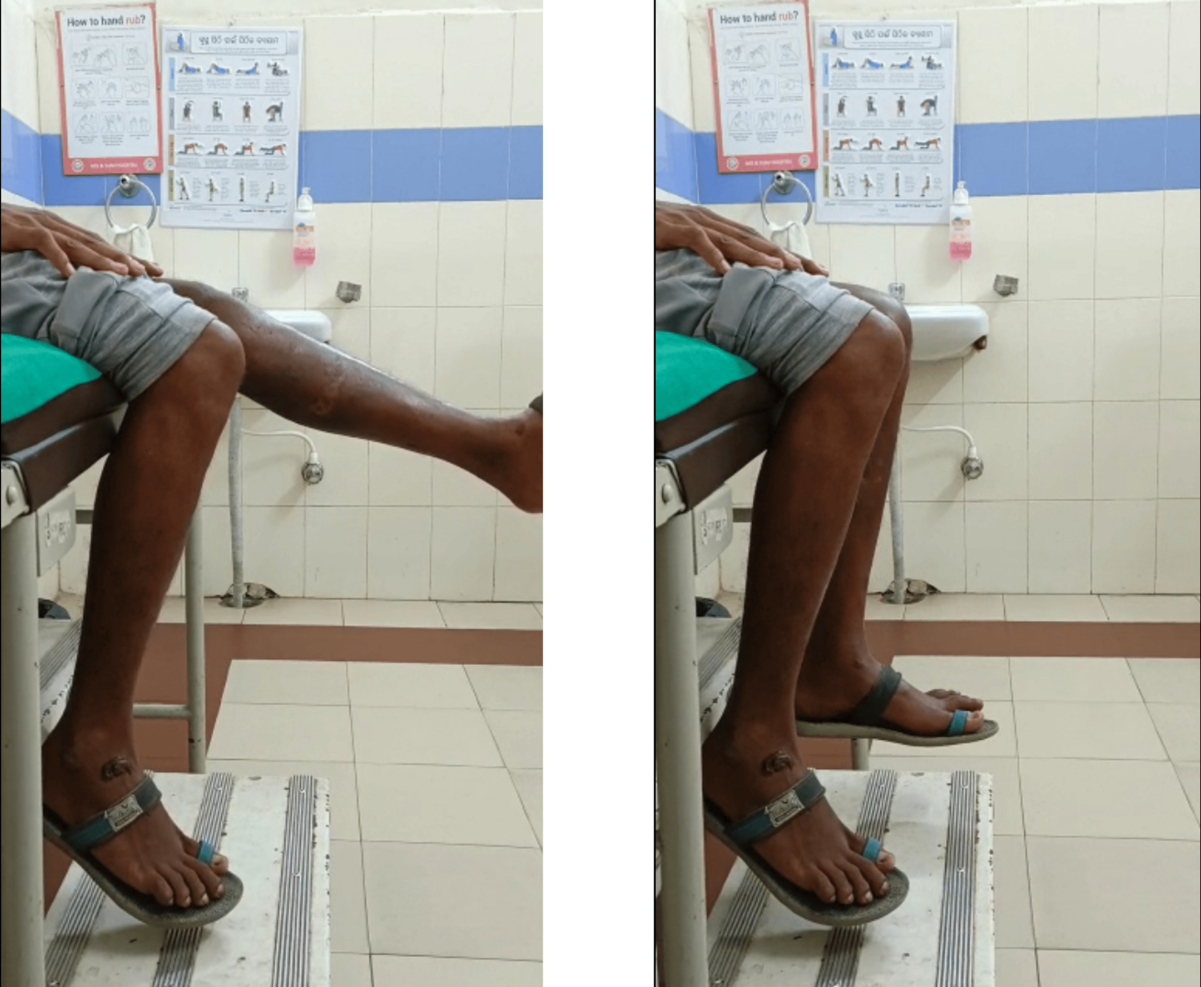
Knee range of motion after Ilizarov fixator removal

Clinical knee ROM was also checked after Ilizarov frame removal as shown in Figure 5.

Limitations of the study

Our study did have a few limitations. Firstly, it was a single-centre study with a small sample size. Secondly, all the patients chosen for the study were surgically managed by more than one surgeon. Finally, a longer follow-up period could have been used for observing any further complications, both surgical and non-surgical.

## Conclusions

The Ilizarov ring fixator is an excellent device to aid in union of the fracture fragments in proximal tibia fractures. Early knee bending and mobilization of the patient aids in micromotion of the fracture site which in turn aids in early fracture site union and increases the morale of the patient.

The average time of fracture site union depends on several factors like degree of commination at the fracture site, presence or absence of infection, co-morbid conditions, smoking, and the fracture type. Patient compliance does play a key role in both functional and radiological outcomes in a successful union at the fracture site.

## Appendices

ASAMI scoring

**Table 6:** Association for the Study and Application of the Methods of Ilizarov (ASAMI) Scoring System Source: Hussain et al., 2008 [22]; Creative Commons Attribution-NonCommercial 2.0 Generic License (CC BY-NC 2.0)

Bone results	
Excellent	Union, no infection, deformity<7°, limb length discrepancy<2.5 cm
Good	Union + any two of the following: no infection, deformity<7°, limb length discrepancy<2.5 cm
Fair	Union +only one of the following: no infection, deformity<7°, limb length discrepancy<2.5 cm
Poor	Non-union / refracture / union + infection + deformity>7° + limb length discrepancy>2.5 cm
Functional results	
Excellent	Active, no limp, minimum stiffness (loss of <15°knee extension/<15°dorsiflexion of ankle), no reflex sympathetic dystrophy, insignificant pain
Good	Active with one or two of the following: Limp, stiffness, RSD, significant pain
Fair	Active with three or all of the following: Limp, stiffness, RSD, significant pain
Poor	Inactive (unemployment or inability to return to daily activities because of injury)
Failure	Amputation
